# Single-cell sequencing and machine learning-based prediction of spliceosome-associated factor 2 may represent potential targets for osteoarthritis

**DOI:** 10.1016/j.ocarto.2026.100798

**Published:** 2026-04-18

**Authors:** Baihui Yang, Xiangde Li, Yiji Su

**Affiliations:** a The First Clinical Medical College, Guangxi Medical University, No. 22 Shuangyong Road, Nanning, Guangxi, China; b Department of Radiation Oncology, The Second Afliated Hospital of Guangxi Medical University, No. 166 East University Road, Nanning, Guangxi, China; c Department of Rehabilitation Medicine, The First Affiliated Hospital of Guangxi Medical University, No. 6 Shuangyong Road, Nanning, Guangxi, China; d Department of Rehabilitation Medicine, The First Affiliated Hospital of Guangzhou Medical University, No. 151 Riverside Road, Guangzhou, Guangdong, China

**Keywords:** Osteoarthritis, Spliceosome-associated factor, Single-cell sequencing, machine learning

## Abstract

**Background:**

Osteoarthritis has become a global health challenge due to its complex pathologic mechanisms. Spliceosome-associated factor 2 (SYF2) has been reported in tumors and neurological diseases, but not in studies of osteoarthritis. We employed single-cell sequencing and machine learning techniques to predict SYF2 as a potential therapeutic target for osteoarthritis and the underlying mechanisms involved.

**Methods:**

Single-cell dataset (GSE220243), cartilage tissue gene expression profiles (GSE169077, GSE117999, GSE53857) and blood sample expression profile (GSE48556) were obtained. We combined single-cell sequencing analysis and machine learning to sort candidate targets for osteoarthritis. We used GSEA analysis to predict the mechanisms of core target for osteoarthritis, and ultimately established osteoarthritis animal models to validate the screened targets.

**Results:**

Bioinformatics screening revealed a negative association between SYF2 and osteoarthritis. GSEA analysis showed that SYF2 negatively correlated with apoptosis. After establishing an osteoarthritis animal model, relative mRNA and protein expression levels were measured, consistent with the bioinformatics prediction results.

**Conclusions:**

Our research identified a previously unreported potential target for osteoarthritis, SYF2, through single-cell sequencing and machine learning. This target is likely to be related to cell apoptosis.

## 1 Introduction

Osteoarthritis is a common chronic disease associated with progressive joint dysfunction. The main pathological feature of osteoarthritis is progressive degeneration of articular cartilage. Based on the causative factors, OA can be classified into primary OA and secondary OA, with primary OA being the most common type [1,2]. Symptoms of arthritis include joint pain, swelling and restricted mobility. The incidence of age-related arthritis is increasing year by year globally and is rapidly increasing after age 50, becoming one of the leading causes of disability in more elderly persons [3,4]. It poses a significant economic and psychological burden not only on patients and their families, but also on socio-economic and health systems [5,6]. Oral anti-inflammatory drugs, analgesics and physiotherapy are the main forms of OA treatment. However, each has its own limitations [[7], [8], [9]]. Despite significant advances in the research and treatment of osteoarthritis, the molecular mechanisms underlying its onset and progression require further investigation. Therefore, it is particularly important to investigate the pathogenesis and progression of osteoarthritis and identify potential therapeutic targets.

Traditional bulk RNA-seq sequencing can detect the average expression levels of genes across large numbers of cells or tissues, but it struggles to reveal the cellular heterogeneity within tissues [10]. Compared with bulk RNA-seq sequencing, single-cell RNA sequencing revealing the hidden complexity and diversity of cell populations, this technique is important for studying potential therapeutic targets and mechanisms of disease onset and progression [11,12]. Lv et al. investigated the role of iron death-associated chondrocytes in the pathogenesis of osteoarthritis (OA) and found that transient receptor potential vanillo1 reduces chondrocyte apoptosis by upregulating the expression of glutathione peroxidase 4 (GPX4) [13]. Osteopontin (OPN) is a protein encoded by SPP1, Qu et al. found a cohort of SPP1-expressing chondrocytes in OA cartilage that exhibited a more pronounced angiogenesis and cellular senescence phenotypes, providing further insight into the role of OPN in OA progression [14]. These findings highlight the importance of scRNA-seq in uncovering cellular functions and molecular mechanisms associated with OA.

Machine learning, a core branch of artificial intelligence, is currently widely used to predict and screen potential targets for new diseases, leveraging its strengths in high-dimensional data processing and nonlinear pattern recognition [15,16]. Zhou et al. used machine learning to identify genes associated with human aging with high OA characteristics and explore their role and mechanisms in OA, providing new insights for early diagnosis of OA [17]. Machine learning, as an advanced data mining engine, is increasingly becoming an important catalyst for accelerating research innovation.

SYF2 is a spliceosome-associated factor that encodes a nuclear protein that interacts with Cyclin-D binding protein 1 [18]. SYF2 depletion reduced breast cancer cell resistance to doxorubicin in tumor research, Iris Tanaka et al. identified a selective splicing pathway that lead to doxorubicin resistance in breast cancer cells [19] , and Tao et al.'s study indicated that downregulation of miR-621 is associated with poor gastric cancer prognosis, and as a tumor suppressor gene, miR-621 targets SYF2 to inhibit cell cycle and gastric cancer cell proliferation [20]. However, no studies have yet reported on the association between SYF2 and OA. In this study, we employed a combined approach of single-cell RNA sequencing with bulk RNA sequencing, machine learning, and experimental validation to identify and validate SYF2 as a potential target for OA. We anticipate that this work will contribute to the advancement of future osteoarthritis research.

## 2 Method

### 2.1 Reagents

RNA purification kit (Cat. K0731) purchased from Thermo Fisher Scientific (USA), reverse transcription kit (Cat. RT203) purchased from YUNBIO (Kunming, China), SYBR qPCR Master mix (Cat. 22,204-1) purchased from TOLOBIO (Shanghai, China), neutral tissue fixative (Cat. G1101) purchased from Servicebio (Wuhan, China), SYF2 Rabbit pAb purchased from Bioss (Beijing, China).

### 2.2 Data download and preprocessing

**Fig. 1:**
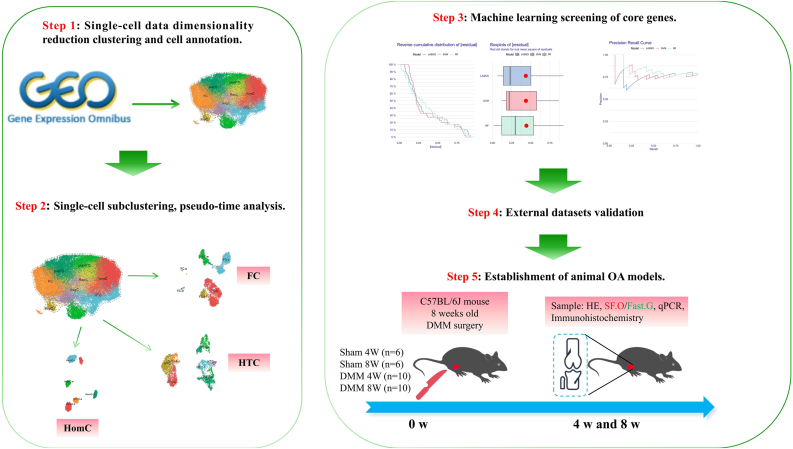
The research process and methodology are illustrated as shown.

Download the publicly available 10X Genomics single-cell RNA transcriptomic map GSE220243 [21] from the GEO database (https://www.ncbi.nlm.nih.gov/geo/). The samples include three normal human knee cartilage samples (GSM6797148, GSM6797149, GSM6797150) and 3 OA knee joint cartilage samples (GSM6797154, GSM6797155, GSM6797156). Low-quality cells were filtered out based on the following criteria: gene feature (nFeature) counts ranging from 300 to 6000, mitochondrial gene proportion <10%, and hemoglobin gene proportion <3% (Supplementary Fig. 1). Ultimately, 37,215 cells were retained for subsequent analysis. Remove batch effects between samples using the harmony function in R software. We visualized the cell clustering results by reducing high-dimensional information to a two-dimensional plane using the uniform manifold approximation and projection (UMAP) technique. Similarly, bulk-RNA sequencing expression profiles of human cartilage tissue (GSE117999, GSE169077), human peripheral blood mononuclear cells (GSE48556) and mouse cartilage tissue (GSE53857) were downloaded from GEO for subsequent analysis. We adopted the same preprocessing method for all bulk-RNA datasets included in the study. Specifically, we first standardized all gene expression values within the expression matrix uniformly, and then replaced the gene probe IDs within the expression matrix with gene names based on the platform file. After completing the data preprocessing, we maintained the above expression matrix files separately to ensure the singularity of tissue types and eliminate batch effects. The overall research workflow of this study is shown in Fig. 1.

### 2.3 Cell annotation

We used the FindAllMarkers function to identify marker genes for each cluster of chondrocytes and annotated the chondrocytes in each cluster based on previous studies[22,23,24]. Marker genes for each chondrocyte subpopulation are shown in Supplementary Table 1.

### 2.4 Subcluster cell annotation

Previous studies have shown that HomC, HTC and FC are concentrated at the beginning and end of the cell differentiation trajectory, respectively [22,23]. Based on the biological process component of the GO enrichment analysis, we inferred the primary biological functions of the subclustered chondrocytes (Supplementary Table 2).

### 2.5 Pseudo-time analysis

Using the monocle package (version 2.30.1), single-cell pseudo-time trajectories were constructed for HomCs, HTCs and FCs, and the differentiation order of cells within each subcluster was inferred [24]. the “DDRTree” algorithm was used to determine cell differentiation trajectories through fully unsupervised learning. By performing gene dynamic analysis on cells along the trajectories, the expression patterns of pseudo-temporal-related genes during cell type transitions were inferred, and genes with significant expression changes were identified. The top 20 pseudo-temporal genes from HomCs, HTCs and FCs were intersected to form a candidate set.

### 2.6 Core gene screening

Download bulk-RNA sequencing expression profile GSE48556 from GEO, GSE48556 contains 33 cases normal peripheral blood mononuclear cell samples and 106 cases peripheral blood mononuclear cell samples from OA patients. The random stratified sampling method was adopted to ensure the balance of the sample. The training set and test set were divided in a ratio of 7:3. Data were analyzed by LASSO regression and candidate genes less impact on results were removed applying the glmnet package with penalty coefficient and 10-fold cross-validation. In the random forest algorithm, we defined the number of decision trees and feature selection (mtry), calculated the error rates of 1–500 decision trees, determined the optimal number of decision trees when the error value was the lowest, used the grid search method to optimize the mtry parameter, and performed 10-fold cross-validation to reduce model overfitting [25]. Support vector machine (SVM) calculates the weight coefficients for each candidate gene and eliminates the candidate genes with the lowest weights through the recursive feature elimination method to obtain the ranking of their importance. At the same time, we measure the model performance by plotting the residual reverse cumulative distribution, the precision-recall curve, and the area under the ROC curve.

### 2.7 Screening of differentially expressed genes (DEGs)

Download bulk-RNA sequencing expression profiles GSE169077 and GSE53857 from GEO as external validation sets. The former included 5 cases of normal knee cartilage and 6 cases of osteoarthritis (OA) human knee cartilage. The latter, which included samples 8 weeks after sham surgery and 8 weeks post-meniscal instability surgery. Using the Limma package (Version 3.56.2), we identified differentially expressed genes (DEGs) with significance threshold of | logFC | > 0 and P < 0.05.

### 2.8 Single gene GSEA analysis

GSEA analysis was performed using the GSE117999 datasets. GSE117999 contains 10 cases normal knee cartilage and 10 cases knee cartilage from OA patients. Samples were divided into high and low expression groups using the median of the core gene expression values obtained as a threshold. Perform differential gene analysis using the limma package and calculate the t-statistic and signal-to-noise ratio. Given that the t-statistic in the limma package is more robust than the signal-to-noise ratio, and the sample size of the GSE117999 dataset is relatively small, the differentially expressed genes between the two groups were sorted in descending order based on the t-statistic and according to the method of gene set permutation, as a preprocessing step before the enrichment analysis [26,27]. Gene Ontology (GO) enrichment analysis were performed to infer pathways associated with core genes.

### 2.9 Construction of animal OA models

The research programme has been approved by the Guangxi Medical University Animal Ethics Committee (No. 202411026). The 7-week-old male C57BL/6J mice (20–25g) were purchased from Sibeifu Biotechnology Co., (Beijing, China). The mice were marked in a blinded manner. Specifically, we used randomly numbered ear tags to mark the mice. During the marking process, the experimenters were unaware of the groups to which the mice belonged. After a week of acclimation in conditions of 23°C −25 °C, 40%–60% relative humidity, the right posterior knee capsule was incised open under anesthesia and perform meniscus instability (DMM) surgery. The mice were randomly divided into four groups according to their ear tag numbers and their initial quantity were determined: the sham 4-weeks group (n = 6), the sham-8 weeks group (n = 6), the DMM 4-weeks group (n = 10), and the DMM 8-weeks group (n = 10). The medial meniscus of the knee was not damaged in sham 4-weeks group and the sham 8-weeks group, and the medial meniscus was damaged in the DMM 4-weeks group and DMM 8-weeks group. All mice received intraperitoneal injections of sodium penicillin for three days after surgery to prevent infection. 4 weeks after the DMM surgery, we euthanized the mice in the sham 4-weeks group and the DMM 4-weeks group. 8 weeks after the DMM surgery, we euthanized the mice in the sham 8-weeks group and the DMM 8-weeks group, and their right hindlimbs were collected for follow-up analysis. All mice involved in the study were subjected to periodic body weight monitoring (Supplementary Table 3).

### 2.10 Histopathological analysis

After 3 weeks of knee decalcification, the knee was implanted in paraffin sections, coated with hematoxylin eosin (HE) and safranin O-fast green, sealed with neutral resin glue, and observed and photographed under a pathology microscope (Olympus, Japan).

### 2.11 RT-qPCR

After extraction of knee cartilage from each group, total RNA was extracted according to the kit instructions, cDNA was synthesized by reverse transcriptometry, and real-time fluorescence quantitative PCR analysis was performed by PCR. The amplification conditions were as follows: 95 °C pre-denaturation for 30 s, 95 °C denaturation for 5 s, 60 °C annealed for 35 s, repeated 40 times. Relative gene expression levels in each treatment group were calculated using the 2ˆ-ΔΔCt method. The primer sequences used in this study are shown in Supplementary Table 3.

### 2.12 Immunohistochemical staining

The paraffin sections were treated in the decalcification solution and gradient ethanol, then washed with distilled water. The antigen retrieval conditions were 5min at medium heat in an EDTA solution (pH = 9.0) with microwave heating, followed by 5min of cooling and 10min of low heat. After blocking endogenous peroxidase, they were incubated with 3% BSA for 30 min, and then incubated with SYF2 Rabbit pAb (dilution 1:50) overnight at 4 °C. After washing with PBS (pH = 7.4), they were incubated with goat anti-rabbit secondary antibody for 50min, then stained with DAB, re-stained the cell nuclei, treated with gradient ethanol and dehydrated and sealed, and then observed using a pathological microscope (Olympus, Japan).

### 2.13 Statistical analysis

Statistical analysis and data calculation for the bioinformatics section were studied using R software (version 4.3.1) and animal treatment groups were compared using ANOVA in GraphPad Prism software (version 10.5.0). The experiment should be repeated at least 3 times. P < 0.05 indicated statistical significance.

## 3 Results

### 3.1 Heterogeneity of chondrocytes

**Fig. 2:**
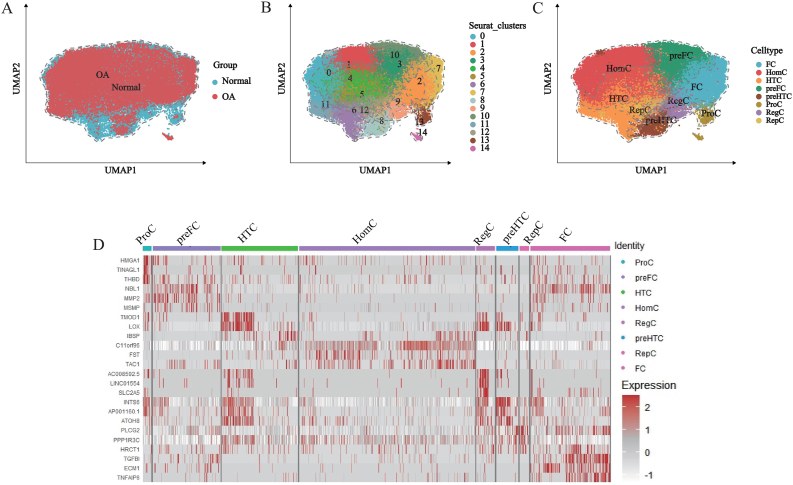
Dimension-reduced clustering and cell annotation of 6 cartilage tissue samples. (A) UMAP plot generated by sample grouping; (B) Dimension-reduced clustering at a resolution of 0.7 yielded 15 cell clusters; (C) Cell annotation identified 8 cartilage cell subpopulations; (D) Top 3 marker genes for each cartilage cell subpopulation.

By generating a clustering tree, we ultimately determined that the optimal dimension reduction clustering parameter was resolution = 0.7 (Supplementary Figs. 2–3). Using the umap algorithm to visualize the chondrocyte data, 37,215 chondrocytes (including 20,526 normal chondrocytes and 16,689 OA chondrocytes) were divided into 15 independent cell clusters (Fig. 2A and B). Based on marker genes, the 15 cell clusters were annotated as homeostasis chondrocytes (HomC), hypertrophic chondrocytes (HTC), pre-fibrocartilage cells (preFC), fibrocartilage cells (FC), regulatory chondrocytes (RegC), prehypertrophic chondrocytes (preHTC), proliferative chondrocytes (ProC), and reparative chondrocytes (RepC)—a total of 8 subpopulations (Fig. 2C), and visualize the top 3 marker genes (Fig. 2D). At the same time, our cell ratio analysis showed that there was no significant difference in the composition of chondrocyte subgroups between the normal group and the OA group (Supplementary Fig. 4A).

### 3.2 Heterogeneity, pseudo-time analysis HomCs

**Fig. 3:**
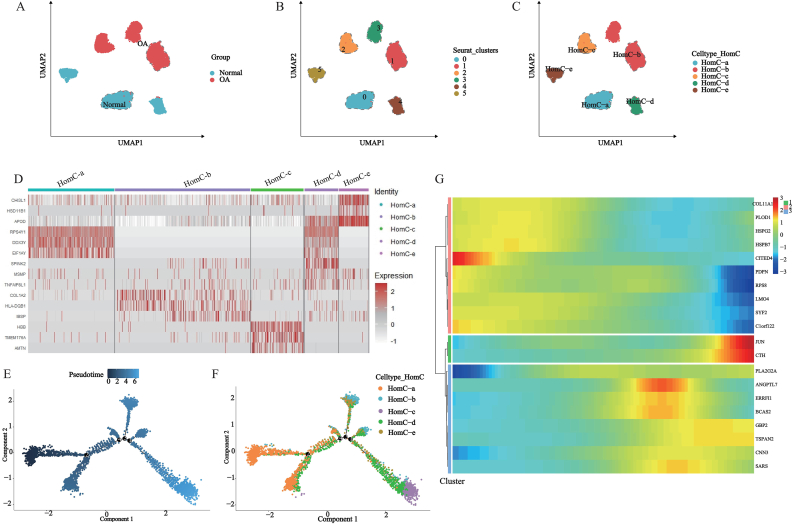
Subcluster pseudo-time analysis in HomC. (A–C) HomC secondary dimensionality reduction clustering and cell annotation; (D) Top 3 marker genes for each HomC cluster; (E–F) Generation of pseudo-time cell trajectories for HomC to identify cell differentiation nodes; (G) Identification of HomC TOP20 pseudo-time genes.

To investigate heterogeneity among HomCs, we subcluster 14,265 HomCs to obtained 6 independent cell subclusters and deduced the primary biological functions of each subcluster, annotated them with HomC-a, HomC-b, HomC-c, HomC-d, and HomC-e based on their primary biological functions (Fig. 3A–C, Supplementary Fig. 4B). We identified marker genes for each cellular subcluster and visualized the top 3 marker genes (Fig. 3D–Supplementary Table 2).

Pseudo-temporal analysis was performed on 5 different types of HomCs to construct cell trajectories. HomC-a is located at the beginning of the cell trajectory, HomC-b and HomC-e at the middle of the cell trajectory and HomC-c at the end of the cell trajectory (Fig. 3E and F). Identification of genes with the top 20 pseudo-temporal genes in HomCs and their expression patterns were divided into 3 modules (Fig. 3G).

### 3.3 Heterogeneity, pseudo-time analysis HTCs

**Fig. 4:**
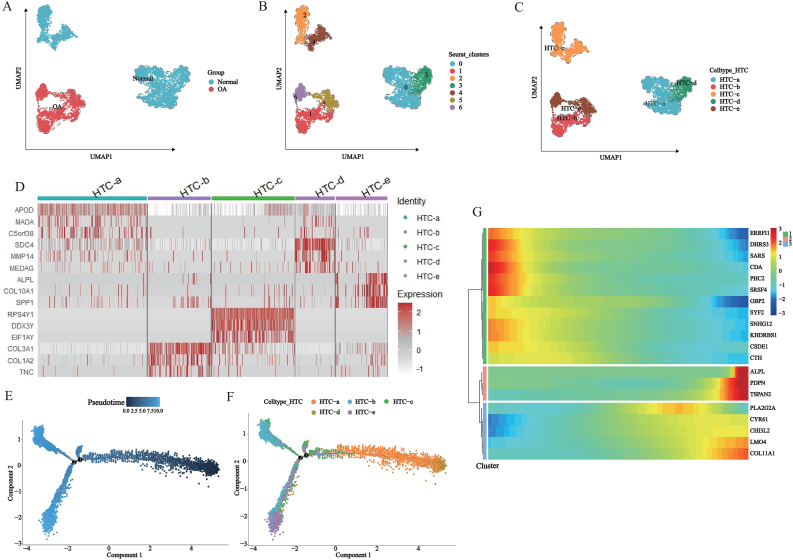
Subcluster pseudo-time analysis in HTC. (A–C) Secondary dimensionality reduction clustering and cell annotation of HTC; (D) Top 3 marker genes for each HTC cluster; (E–F) Generation of pseudo-time cell trajectories for HTC, identifying cellular differentiation nodes; (G) Identification of HTC TOP20 pseudo-time genes.

We obtained 7 independent cell subclusters were obtained after subclustering 6193 HTCs. The primary biological functions of each subcluster were inferred from GO enrichment analysis and annotated based on their primary biological functions such as HTC-a, HTC-b, HTC-c, HTC-d, HTC-e (Fig. 4A–C, Supplementary Fig. 4C). Marker genes were identified for each cell subcluster and the top 3 were visualized (Fig. 4D–Supplement table 2).

Pseudo-temporal analysis was performed on 5 different types of HTCs to construct cell trajectories. HTC-a and HTC-d were at the beginning of the cell trajectory, HTC-c at the middle of the cell trajectory and HTC-e at the end of the cell trajectory (Fig. 4E and F). The top 20 pseudo-temporal genes in HTCs were identified and divided into 3 modules based on their expression patterns (Fig. 4G).

### 3.4 Heterogeneity, pseudo-time analysis FCs

**Fig. 5:**
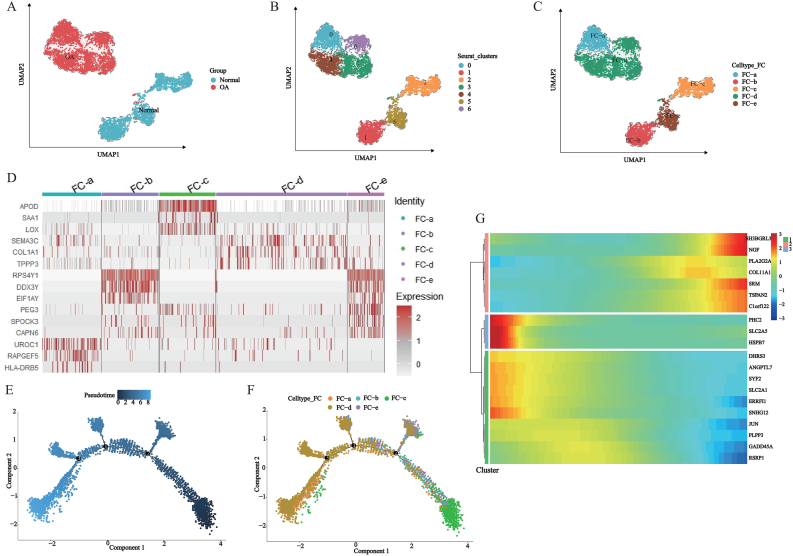
Pseudo-time analysis in FC subclusters. (A–C) Secondary dimensionality reduction clustering and cell annotation of FC; (D) Top 3 marker genes for each FC cluster; (E–F) Pseudo-time cell trajectories generating FC, identifying cellular differentiation nodes; (G) Identification of FC TOP20 pseudo-time genes.

After 6461 FCs, 7 separate subclusters were formed. The main biological functions of each subcluster were extrapolated from GO enrichment analysis and annotated as FC-a, FC-b, FC-c, FC-d, FC-e according to its main biological functions (Fig. 5A–C, Supplementary Fig. 4D). Marker genes were identified for each cell subcluster and the top 3 were visualized (Fig. 5D–Supplementary Table 2).

Pseudo-time analysis was performed on 5 different types of FCs to construct cell trajectories. FC-c was distributed at the beginning of the cell trajectory, FC-b and FC-e were distributed in the middle of the cell trajectory, and FC-a and FC-d were distributed in the middle and end of the cell trajectory (Fig. 5E and F). The top 20 genes with the changing expression levels over time within FCs were identified, along with the top 20 pseudo-temporal genes, which were classified into 3 modules based on their expression patterns (Fig. 5G).

### 3.5 Machine learning screening of core genes

**Fig. 6:**
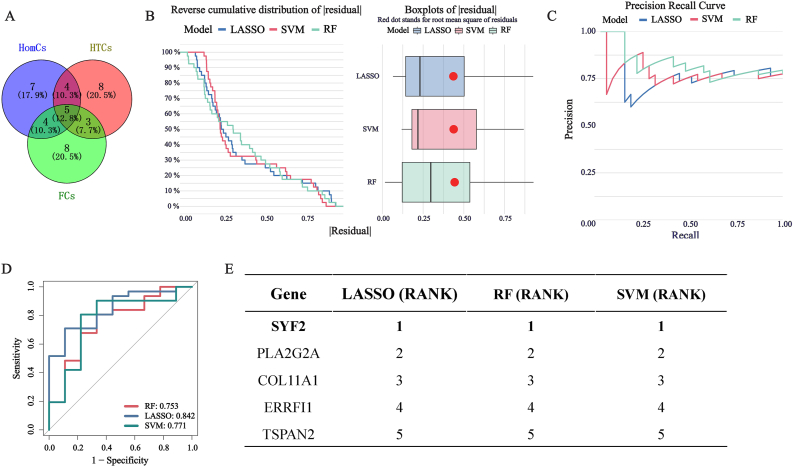
Screening candidate pseudo-time genes using machine learning algorithms. (A) Intersection of pseudo-time genes from HomC, HTC and FC yields 5 candidate genes; (B) The reverse cumulative distribution of the absolute values of model residuals; (C) The precision-recall curve of the prediction models of the machine learning algorithm; (D) A ROC curve was generated, and the AUC area under the curve was calculated to evaluate the model's predictive performance; (E) Sort the candidate genes by their importance.

We identified 5 candidate genes from the intersection of the top 20 pseudo time series genes in HomCs, HTCs and FCs (Fig. 6A, Supplementary Table 4). Included the GSE48556 gene expression profile and employed three machine learning algorithms, namely LASSO regression, RF, and SVM, to screen for core genes. By plotting curves and box plots based on the reverse cumulative distribution of the absolute values of model residuals, we analyzed the model's error. The area under the curve on the left side was smaller, indicating a smaller error of the model. The red dots in the box plot represent the root mean square of residuals, and the smaller the area of the "box” in the box plot, the higher the reliability of the model (Fig. 6B). The area in the lower left corner of the precision-recall curve indicates better prediction accuracy of the model (Fig. 6C). At the same time, the AUC area under the ROC curve was calculated to evaluate the predictive efficacy of the three algorithms. The AUC areas under the ROC curves of RF, SVM, and LASSO were 0.753, 0.771, and 0.842, respectively (Fig. 6D). The candidate genes selected by the three machine learning algorithms were ranked by their importance (Fig. 6E).

### 3.6 External datasets validation and GSEA analysis

**Fig. 7:**
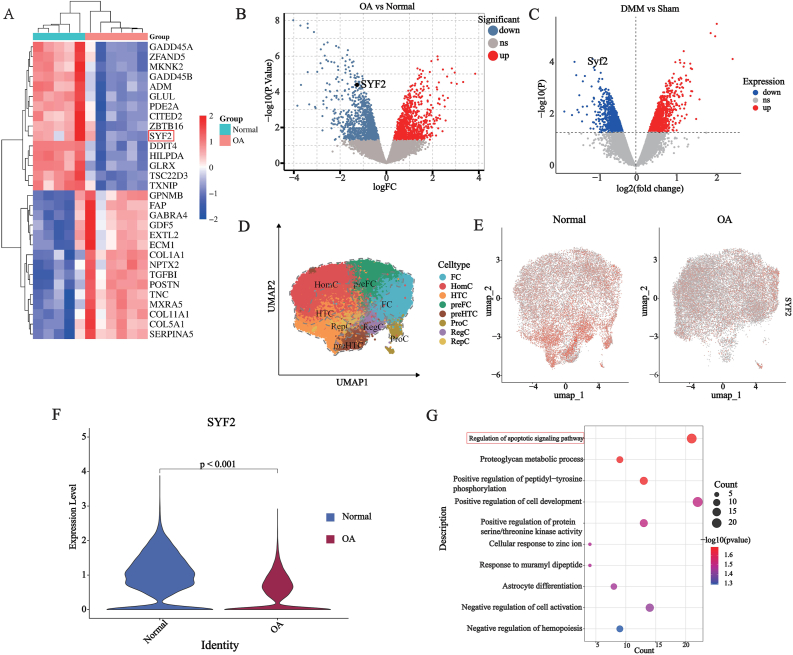
External validation of core genes and enrichment analysis among cell subpopulations. (A–B) External validation datasets GSE169077 indicate SYF2 is downregulated in OA; (C) External validation datasets GSE53857 indicate SYF2 is downregulated in DMM model; (D–F) The single-cell feature map and violin plot indicate that the expression level of SYF2 is generally lower in the OA group compared to the normal group. This difference is more pronounced in specific subgroups of chondrocytes; (G) GO enrichment analysis of the chondrocytes subpopulation with the greatest difference in SYF2 expression between the two groups.

We introduced the GSE169077 and GSE53857 datasets as external validation for differential gene expression analysis and identified SYF2 as a potential target for OA, which was significantly reduced in both human OA cartilage samples and mouse DMM-treated cartilage samples cartilage samples (Fig. 7A–C). Results were consistent with pseudo-time analysis: SYF2 expression levels decrease over time from normal to OA cartilage (Figs. 3G–. 4G and 5G). The clinical diagnostic efficacy of SYF2 against OA was analyzed using the GSE169077 dataset to map the ROC curves and calculate the area under the curve using MMP9 as a positive control (Supplementary Fig. 4E). Our single-cell feature map also indicates that SYF2 has a higher expression level in normal samples, and this is particularly evident in specific chondrocyte subpopulations such as preHTC and HTC (Fig. 7D–F). We extracted the chondrocytes with the greatest expression difference of SYF2 between the two groups. By identifying the DEGs between them and conducting GO enrichment analysis, we found that there were significant differences in apoptosis regulation between these cells (Fig. 7G).

### 3.7 SYF2 expression was significantly reduced in OA model mice

**Fig. 8:**
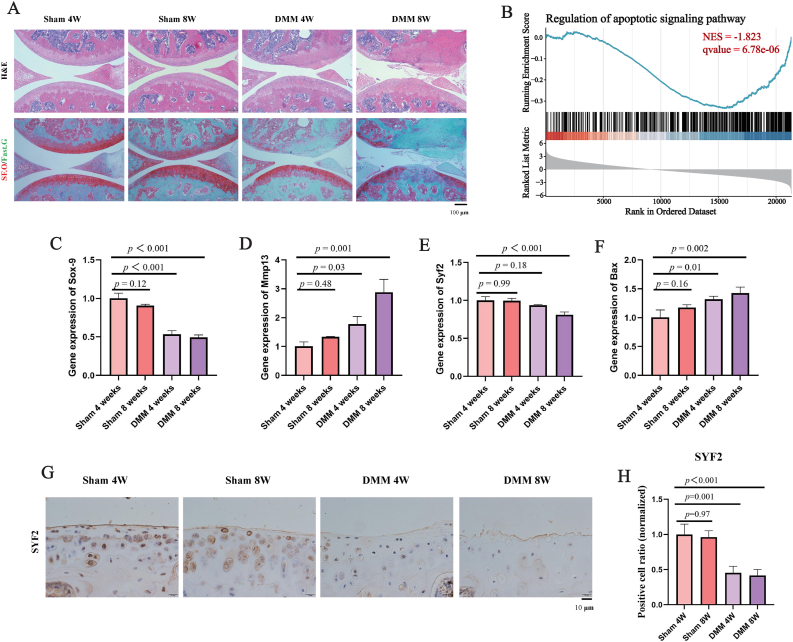
Histopathology, qPCR and immumohistochemical staining Analysis. (A) Hematoxylin and eosin (H&E) and safranin O-fast green staining of knee joints in each treatment group; (B) SYF2 single-gene GSEA analysis; (C–D) Relative expression levels of anabolism and catabolism marker genes in knee joint tissue; (E) Relative mRNA expression level of Syf2 in knee joint tissue. (F) Relative mRNA expression levels of apoptosis marker genes in knee joint tissue. (G–H) SYF2 immunohistochemical analysis (n = 3). The quantitative analysis data are presented as mean ± SD.

Histopathological analysis using hematoxylin-eosin and safranin-green staining revealed that the surface of the knee joint in the sham-operated group was relatively smooth and flat, with well-organized chondrocytes and only minor defects in collagen and extracellular matrix; At 4 and 8 weeks post-DMM surgery, the surface of the knee joint in mice was rough and uneven, with a significant reduction in the number of chondrocytes, collagen, and extracellular matrix, with the most severe changes observed at 8 weeks (Fig. 8A). Single-gene GSEA analysis indicated that SYF2 negatively correlated with apoptosis (NES = −1.823, qvalue = 6.78 × 10^−6^) (Fig. 8B). The results of qPCR showed that DMM surgery significantly inhibited cartilage anabolism in the knee joints of mice and increased catabolism (Fig. 8C and D). At 8 weeks post-DMM surgery, SYF2 expression decreased (Fig. 8E), apoptosis levels increased (Fig. 8F). Immunohistochemical analysis revealed that the relative expression level of the SYF2 protein significantly decreased at 4 weeks and 8 weeks after DMM surgery, the chondrocytes that are positive for SYF2 are mainly concentrated in the superficial layer of the cartilage (Fig. 8G and H). This is consistent with the results of the aforementioned bioinformatics analysis.

## 4 Discussion

Recent studies suggest SYF2 may serve as a broadly effective treatment for amyotrophic lateral sclerosis (ALS). Gabriel R. Linares and colleagues found that inhibition of SYF2 improved neurological dysfunction and motor impairments in mice overexpressing TDP-43 [28]. Xu et al. found that SYF2 was strongly expressed in astrocytes active in the rat cerebral cortex after LPS injection. In vitro studies suggested SYF2 may be involved in neuroinflammation and neuronal apoptosis [29]. Immunohistochemical analysis revealed specific alterations in SYF2 expression among nucleus pulposus cells and inner annular fibrosing cells within degenerative intervertebral disc tissue [30], this suggests that SYF2 may be involved in aging of connective tissue. Currently, there are no reported studies on the association between SYF2 and OA. The pathogenesis of OA was predicted by bioinformatics prediction, qRT-PCR and immunohistochemical staining, suggesting SYF2 may be a potential therapeutic target for OA. Since both cartilage and intervertebral discs are connective tissue, we hypothesized that the mechanism of aging of OA cartilage may be similar to that of intervertebral discs. qRT-PCR analysis revealed a significant decrease in SYF2 expression and upregulation of apoptotic marker genes in cartilage tissue of mice 8 weeks post-DMM surgery. Therefore, we hypothesize that SYF2 may regulate the occurrence and progression of OA through the biological process.

Previous studies have shown that chondrocytes mainly differentiate in two directions during the process: becoming articular cartilage or obtaining a hypertrophic phenotype and becoming HTCs. Under physiological conditions, HTCs are of great significance for bone formation and growth. However, under pathological conditions, the acquisition of a hypertrophic phenotype by chondrocytes is detrimental to the maintenance of cellular homeostasis. At the same time, HTCs will produce matrix metalloproteinases to degrade the cartilage matrix and promote chondrocyte apoptosis [31,32]. Our single-cell sequencing analysis revealed that SYF2 showed more significant differences between the normal group and the OA group in specific chondrocyte subpopulations (such as preHTC, HTC), and enrichment analysis indicated that they had obvious differences in apoptosis regulation, the single-gene GSEA analysis also indicated that SYF2 might be negatively correlated with apoptosis. However, relying solely on single-cell sequencing and GSEA analysis may not be sufficient to predict the causal relationship between SYF2 and apoptosis. Due to the complexity of the biological molecular network, revealing its underlying mechanism may require more evidence. Svinin G et al. reviewed that causal network analysis is a method that helps infer the potential mechanism of regulatory factors in diseases [33]. Tejada-Lapuerta A et al. discussed the application of causal machine learning framework in single-cell transcriptomics and summarized the research progress and prospects of causal models [34]. In the future, we will also consider introducing the above algorithm to further infer the causal relationship between SYF2 and apoptosis in the OA.

After using bioinformatics methods to identify SYF2 as a potential target for OA, we established an in vivo model of OA to verify the previous results. The qPCR results showed that as the OA model after DMM surgery continued to progress, the expression of SYF2 also decreased, and the expression of apoptosis-related marker genes also increased accordingly. Immunohistochemical analysis also indicated that SYF2 was significantly downregulated at 4 and 8 weeks after DMM surgery. However, we found that the expression levels of SYF2 mRNA and protein were not completely consistent. This might be due to the fact that the stability of mRNA is regulated by multiple factors after its generation, and proteins may undergo multiple modifications after translation to fulfill different biological functions, which will directly affect the stability of the proteins. Additionally, there is a temporal difference between the transcription of mRNA and the translation of proteins [[35], [36], [37]]. The limitation of this study lies in the fact that we did not establish a SYF2 knockdown chondrocyte cell line to verify its impact on cell phenotype, nor did we observe the changes in the expression levels of related proteins after SYF2 knockdown. This is also the research content that we need to carry out in the future.

In summary, we screened and validated potential targets through single-cell sequencing, machine learning, and the establishment of an OA animal model. Our study found that the SYF2 may play a protective role in the onset and progression of OA, this effect may be achieved through negative regulation of apoptosis. In-depth research on the specific mechanisms by which SYF2 participates in the onset and progression of OA will conducted in the future.

## Author contributions

Formal analysis, investigation, methodology and writing original draft: Baihui Yang; Writing–review: Xiangde Li; writing–review: Yiji Su. All authors read and approved the final manuscript.

## Data availability statements

All available datasets used in this study were obtained from the GEO (https://www.ncbi.nlm.nih.gov/geo/). More detailed data can be found in the supplementary materials.

## Ethics approval

The datasets used in the bioinformatics section of this study were obtained from publicly available studies in the GEO database and therefore did not require secondary ethical approval. The research protocol for anBimal models was approved by the Guangxi Medical University Animal Ethics Committee (No. 2202411026).

## Disclosure instructions

In preparing this manuscript, the author utilized DeepL for translation and refinement. Following the use of this tool, the author reviewed and edited the content as necessary and assumes full responsibility for the publication's content.

## Conflict of interest

The authors declare that the research was conducted in the absence of any commercial or financial relationships that could be construed as a potential conflict of interest.
