# Supplementary figures and images for: Single-cell sequencing and machine learning-based prediction of spliceosome-associated factor 2 may represent potential targets for osteoarthritis
Source: Osteoarthr Cartil Open. 2026 Apr 18;8(2):100798. doi: 10.1016/j.ocarto.2026.100798 (PMC13141755; doi:10.1016/j.ocarto.2026.100798)

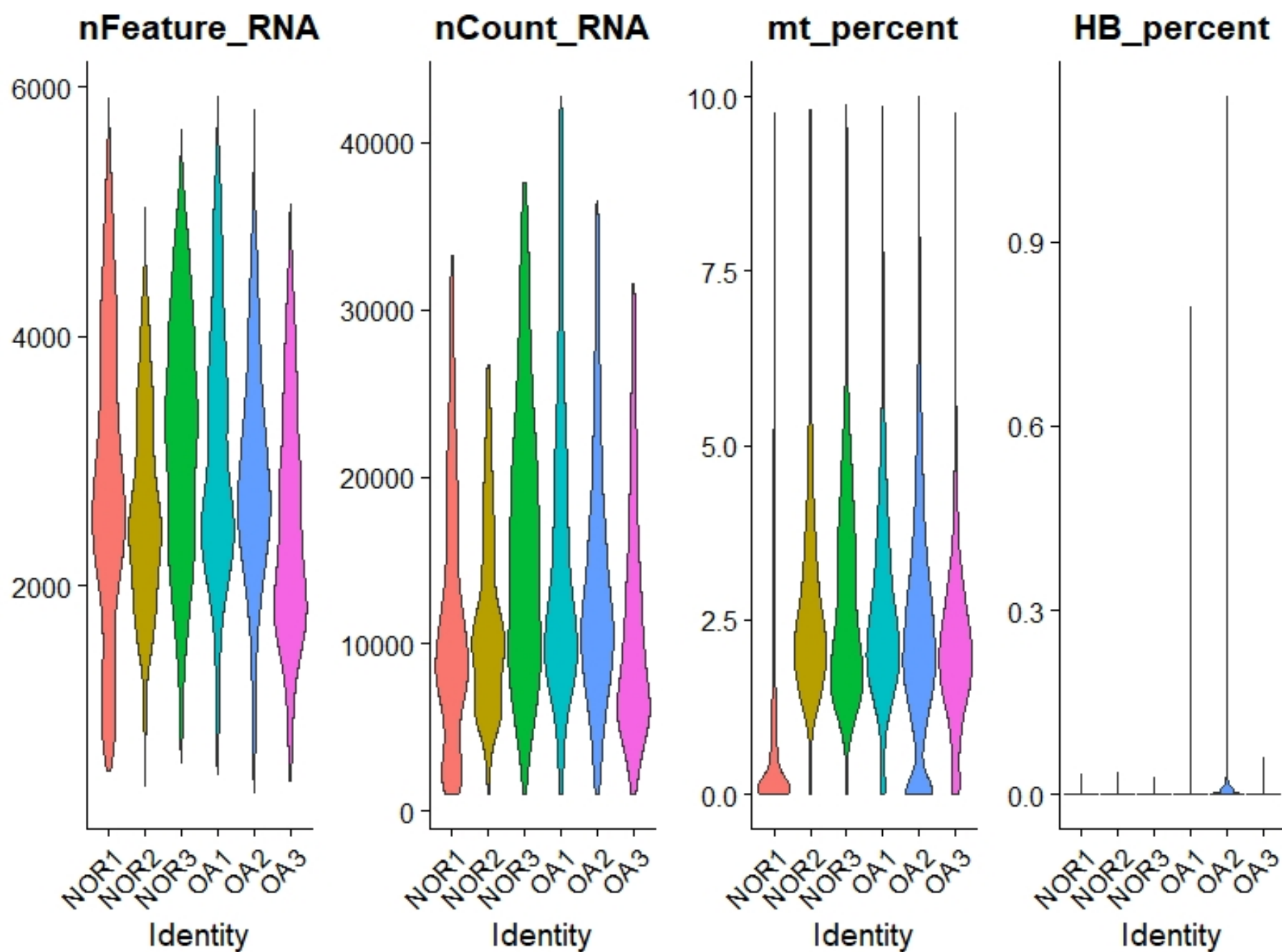

Supplement: Multimedia component 1 [file mmc1.pdf]

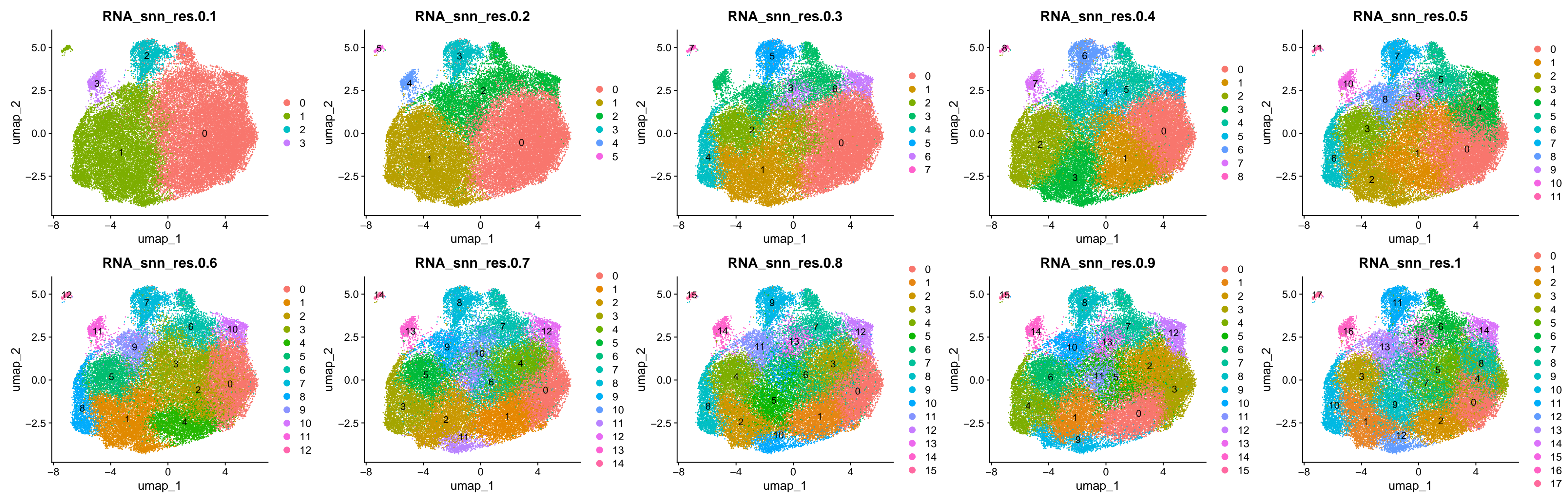

Supplement: Multimedia component 2 [file mmc2.pdf]

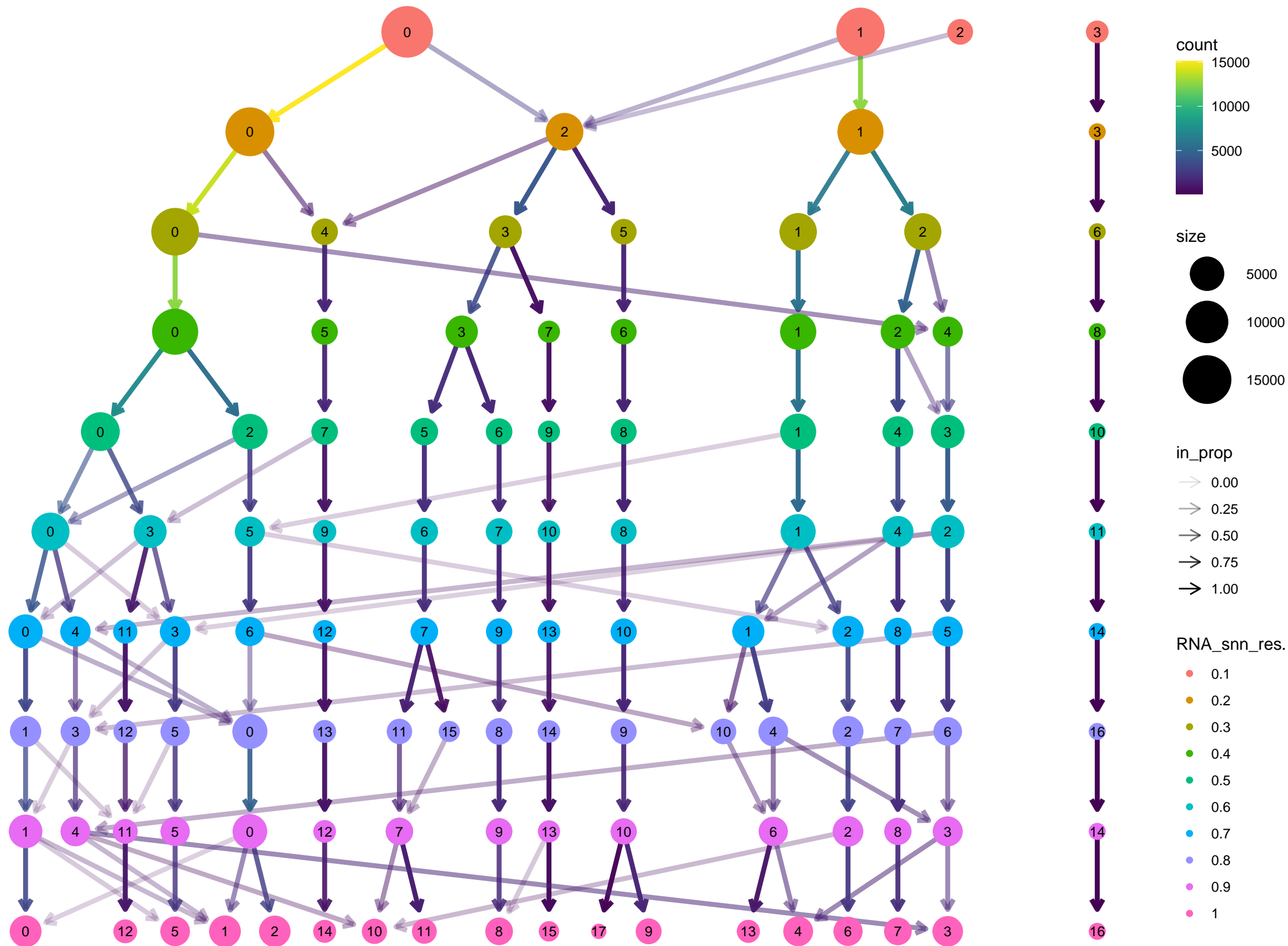

Supplement: Multimedia component 3 [file mmc3.pdf]

A

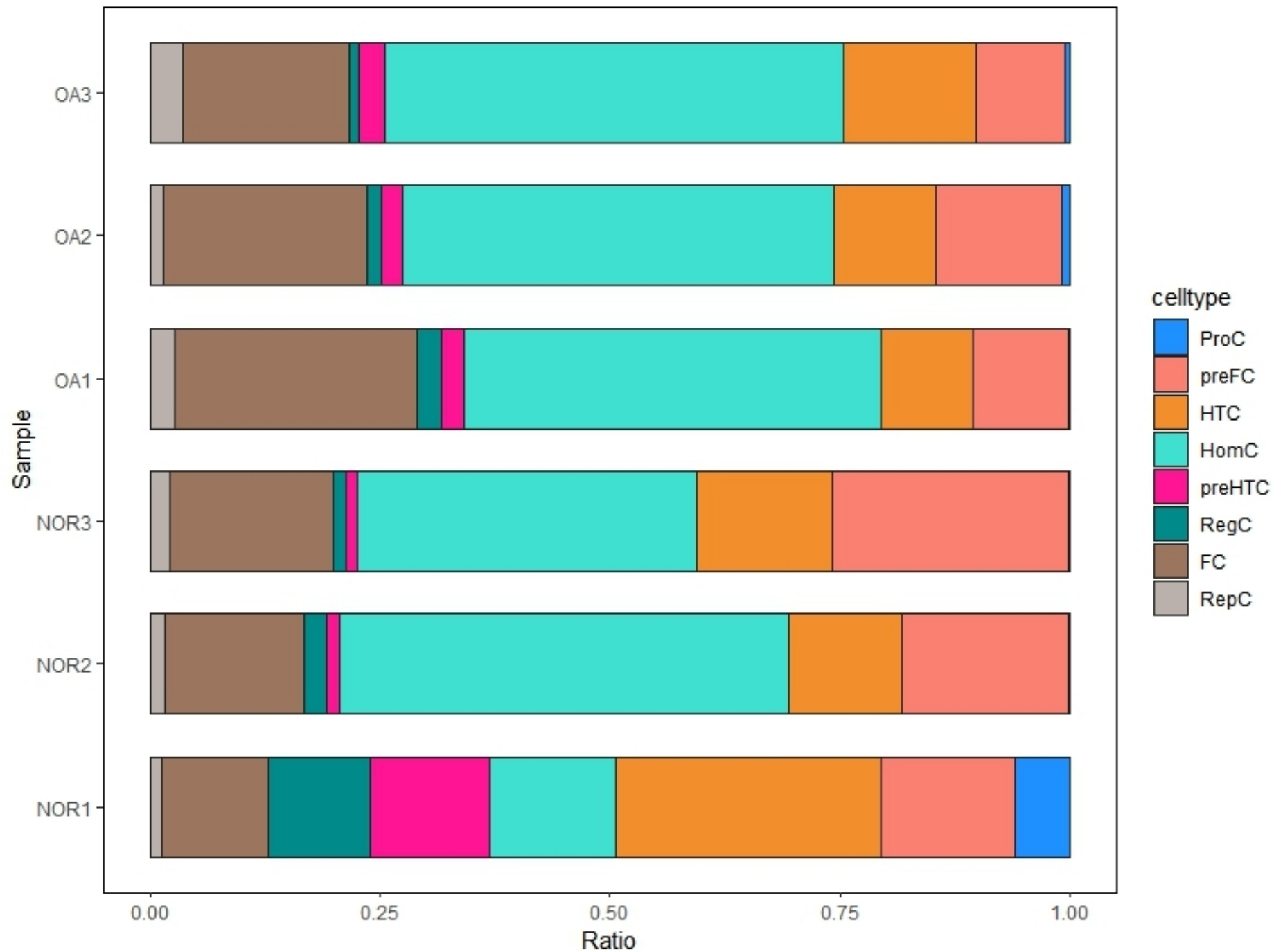

B

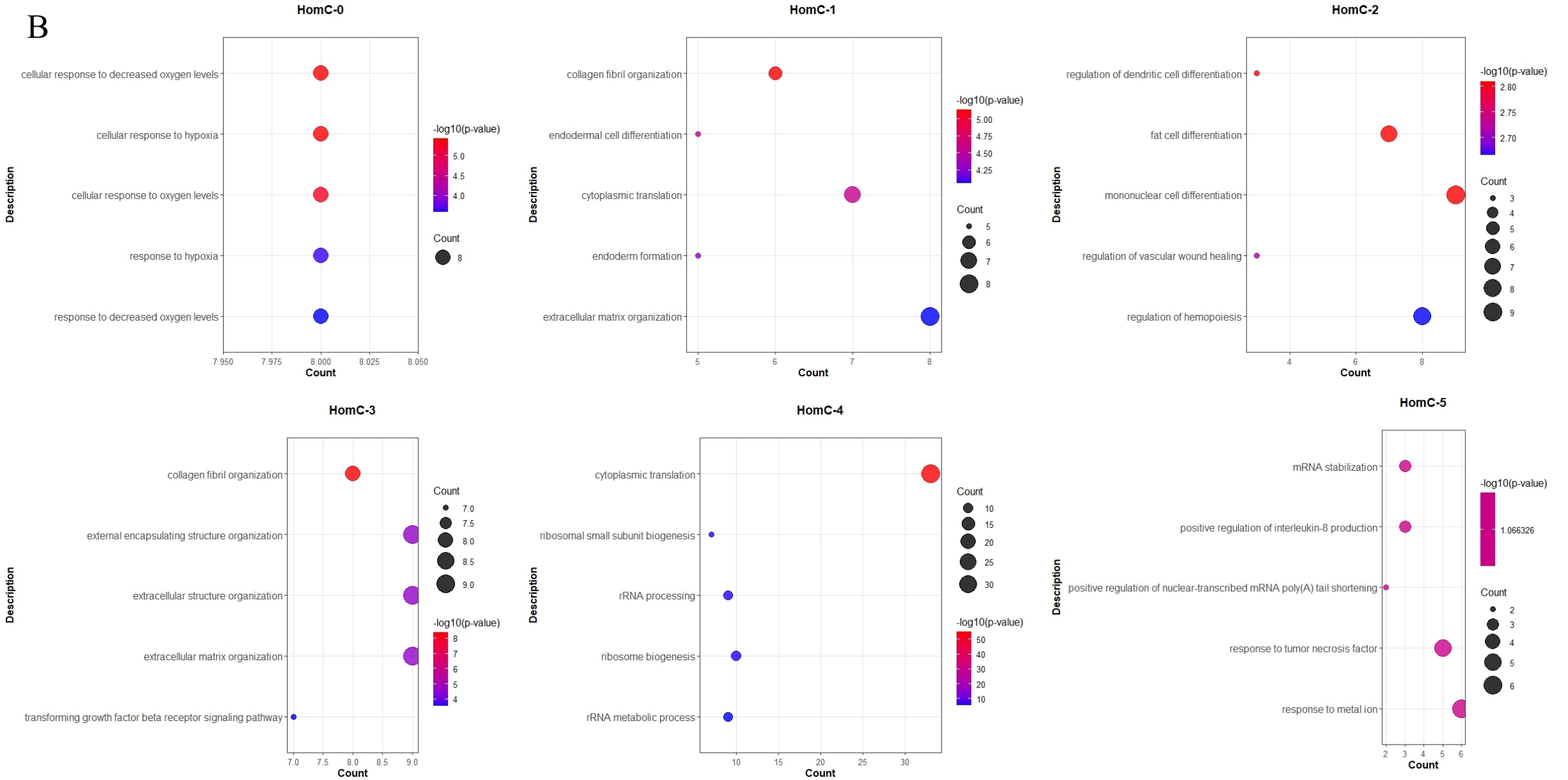

C

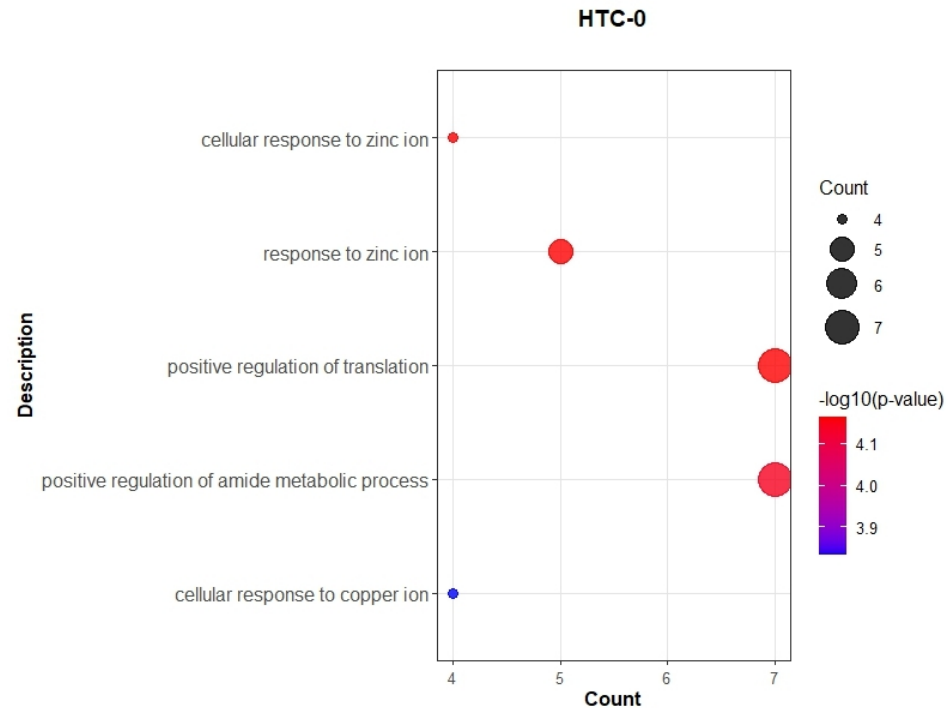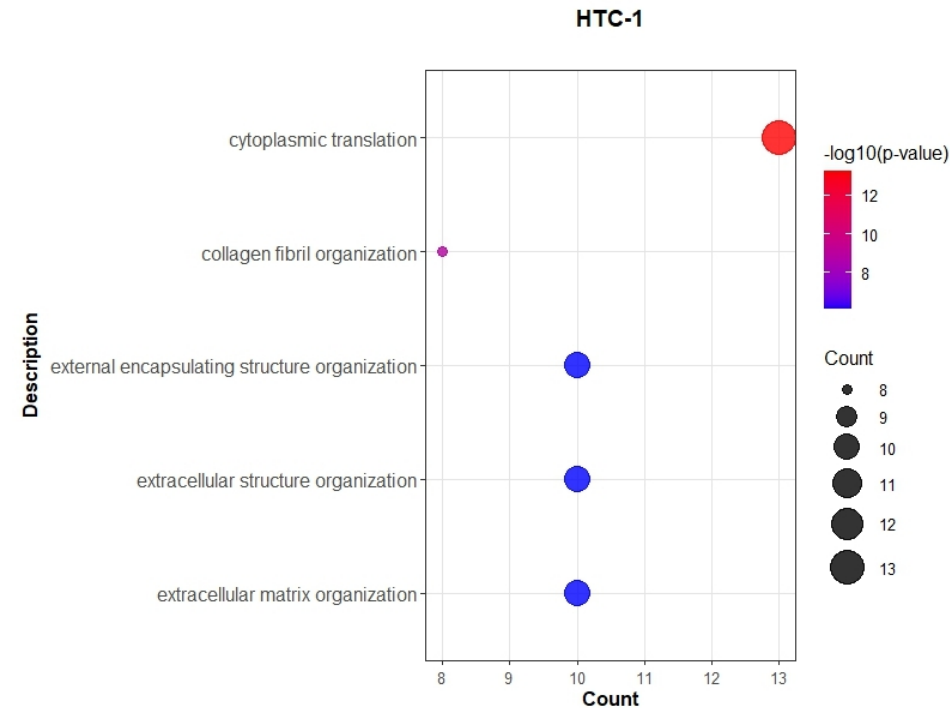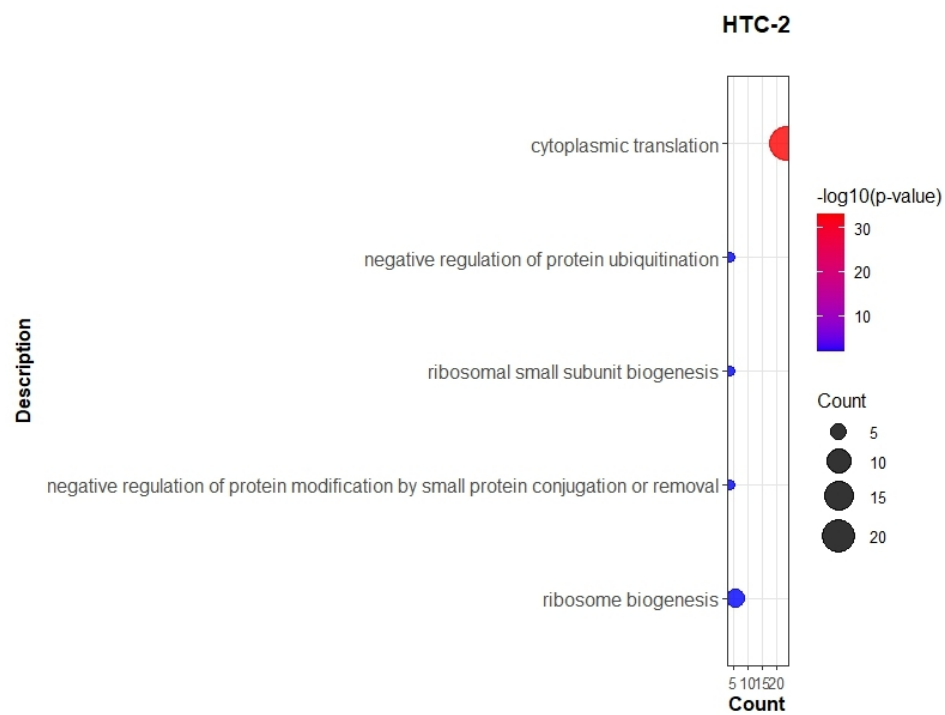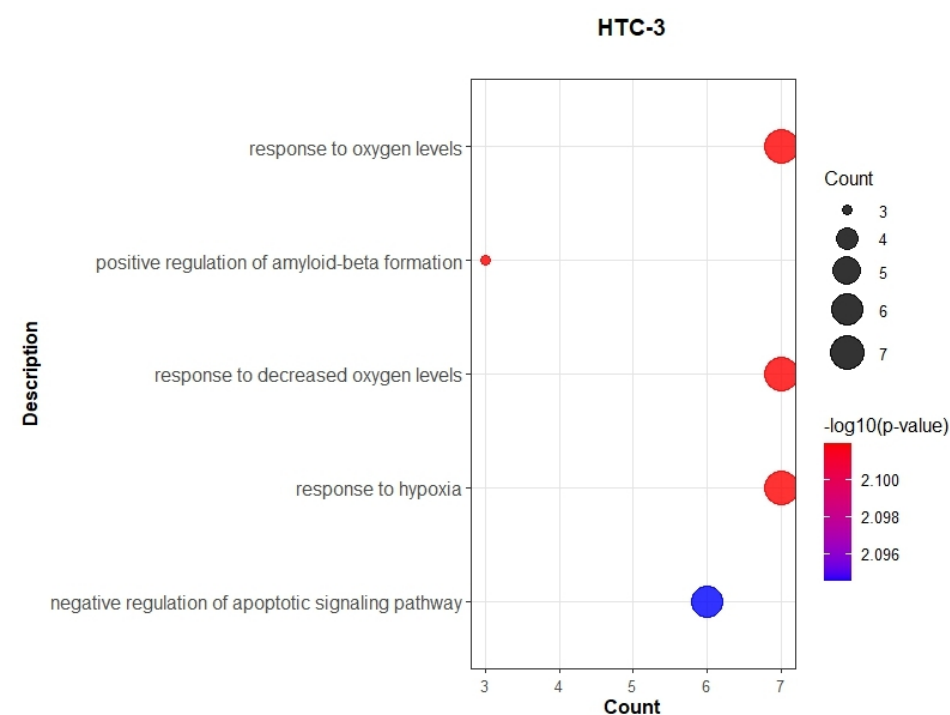

HTC-4

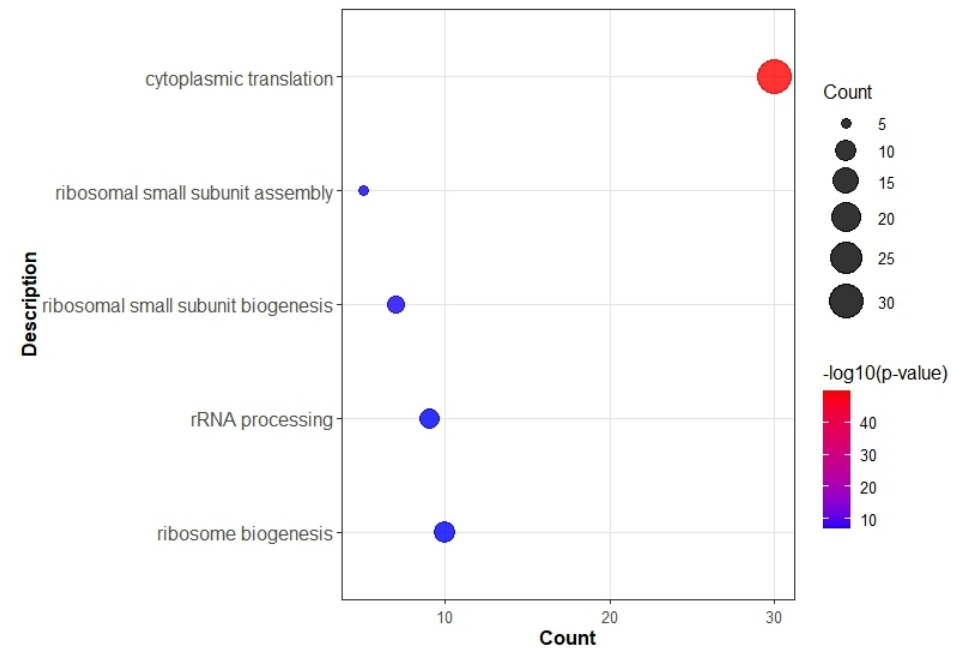

HTC-5

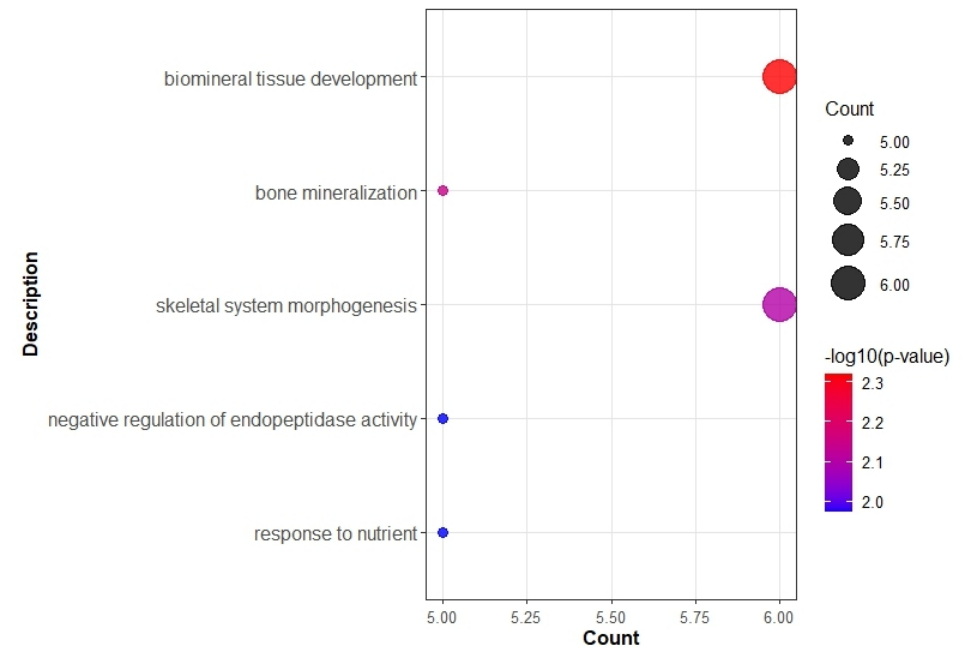

HTC-6

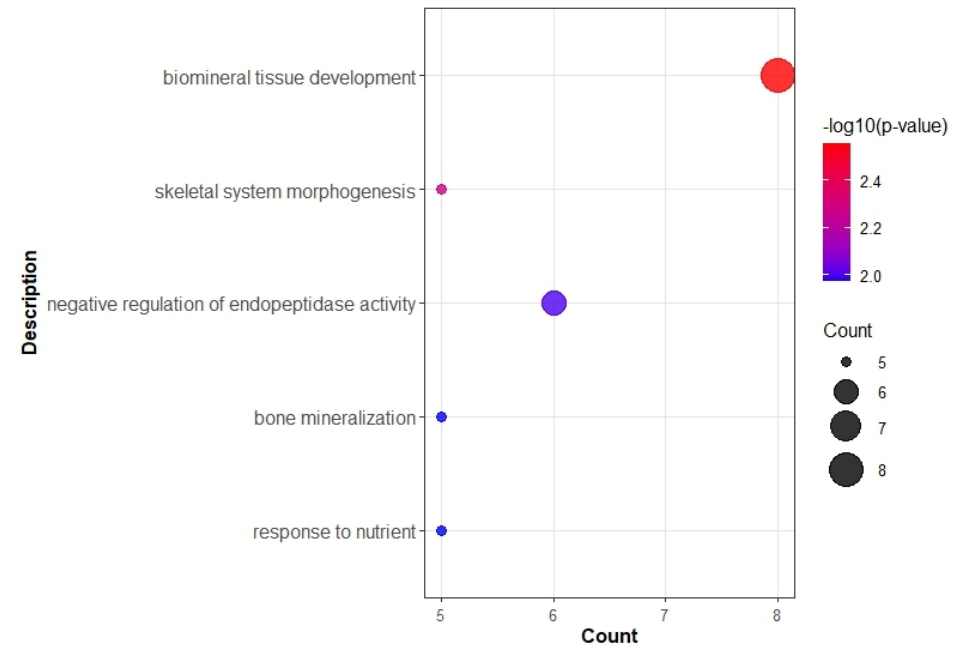

D

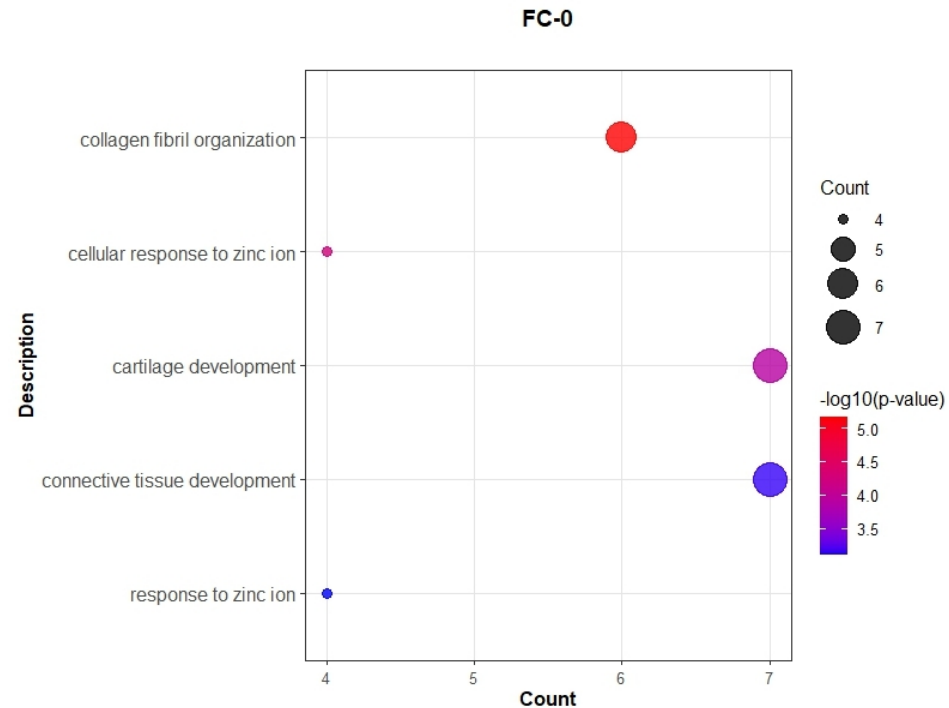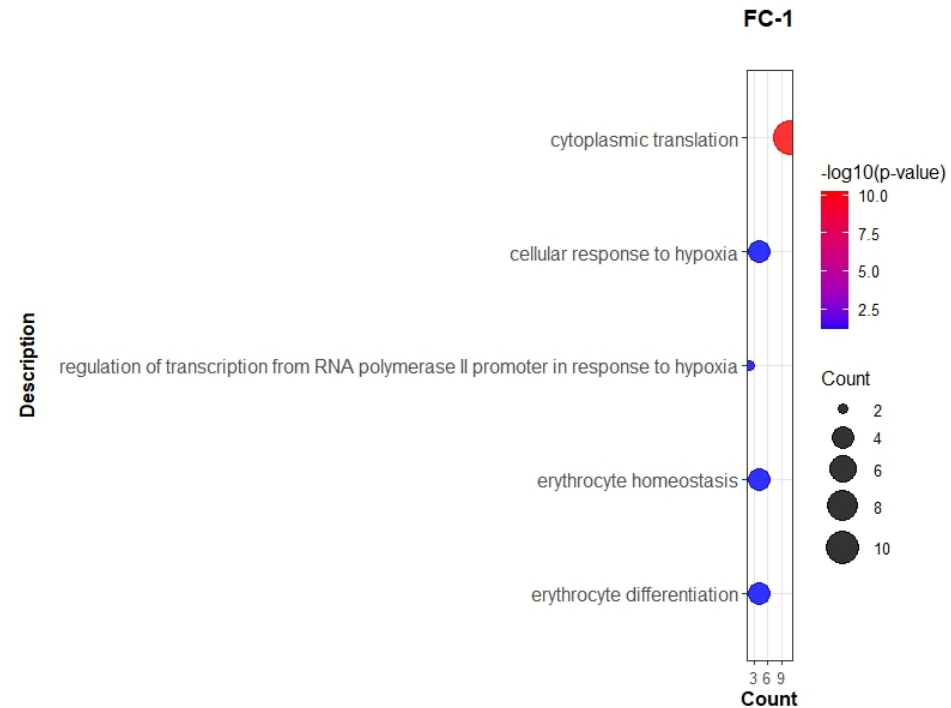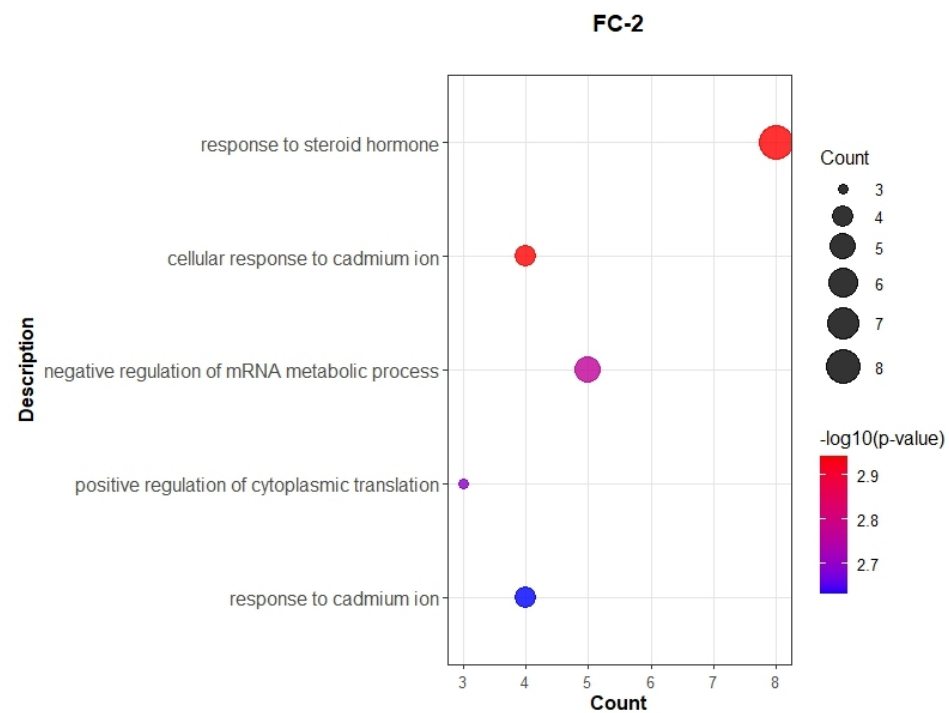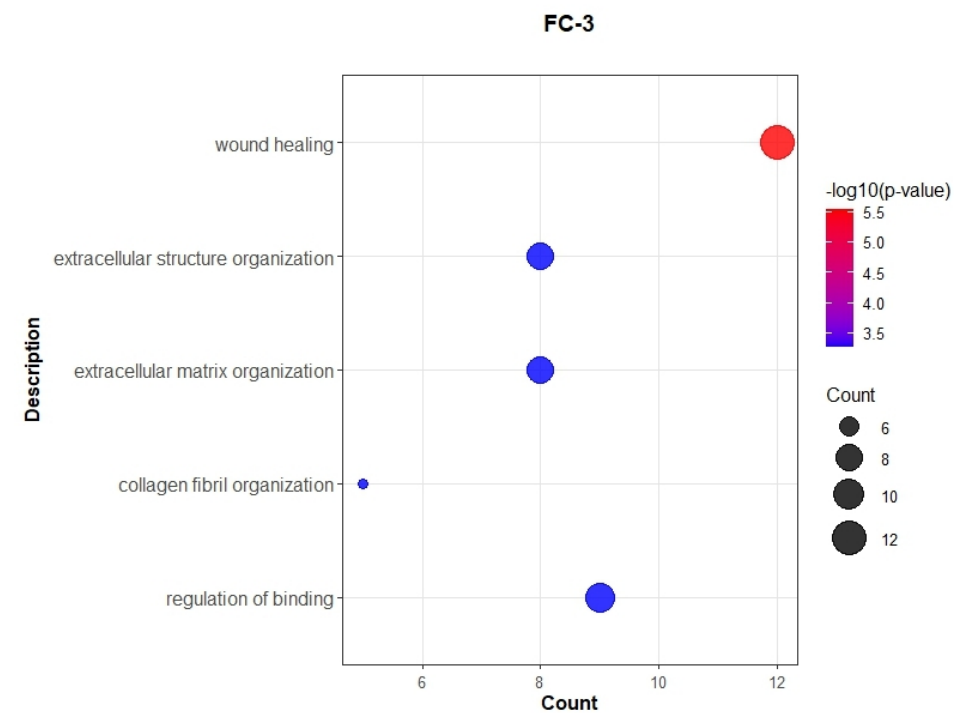

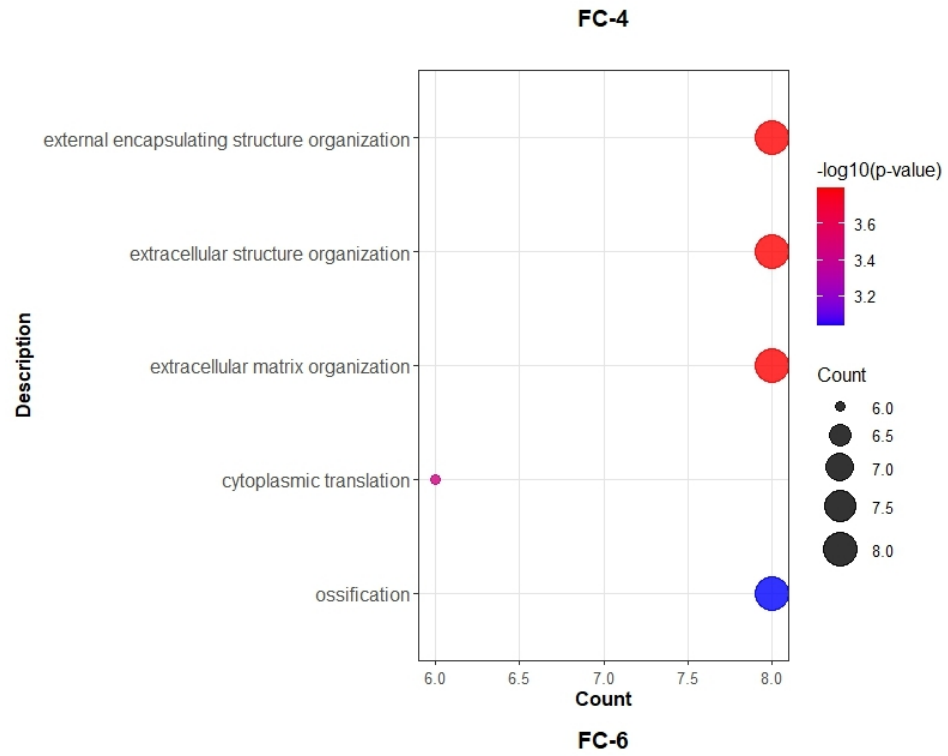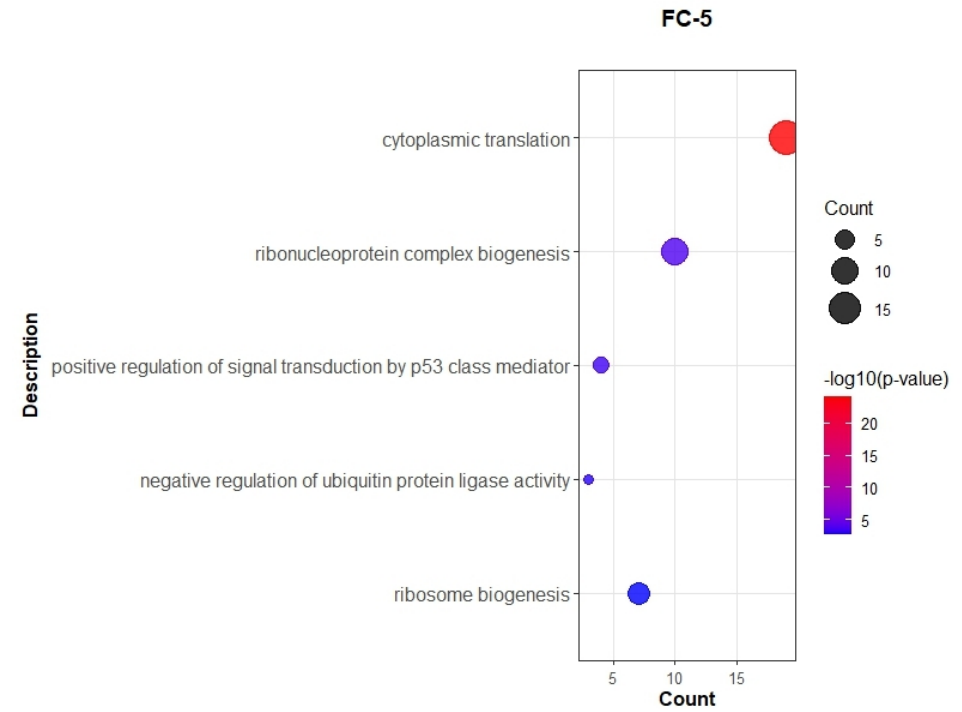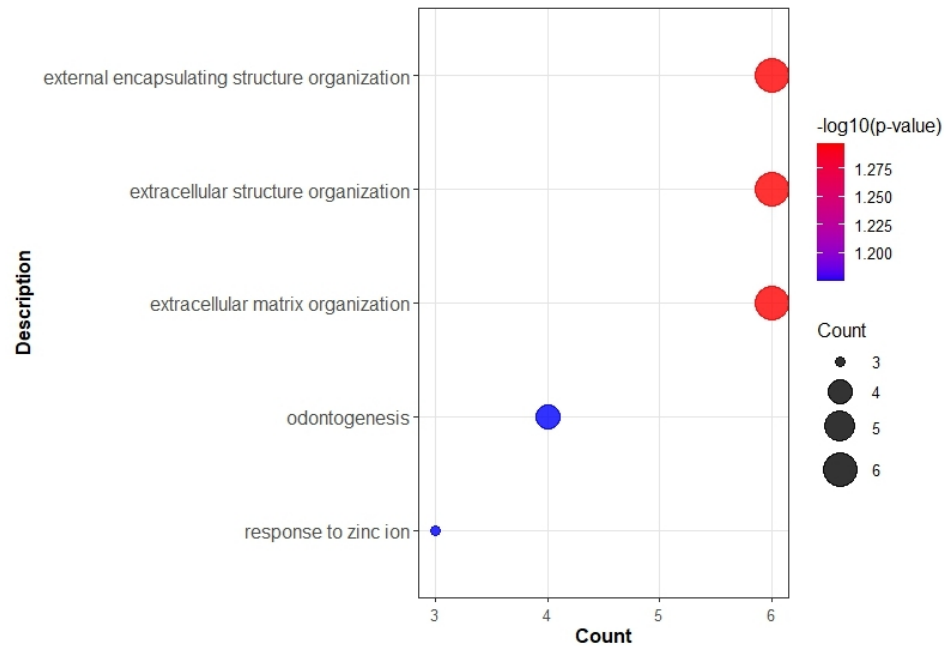

E

**ROC Curve for SYF2**

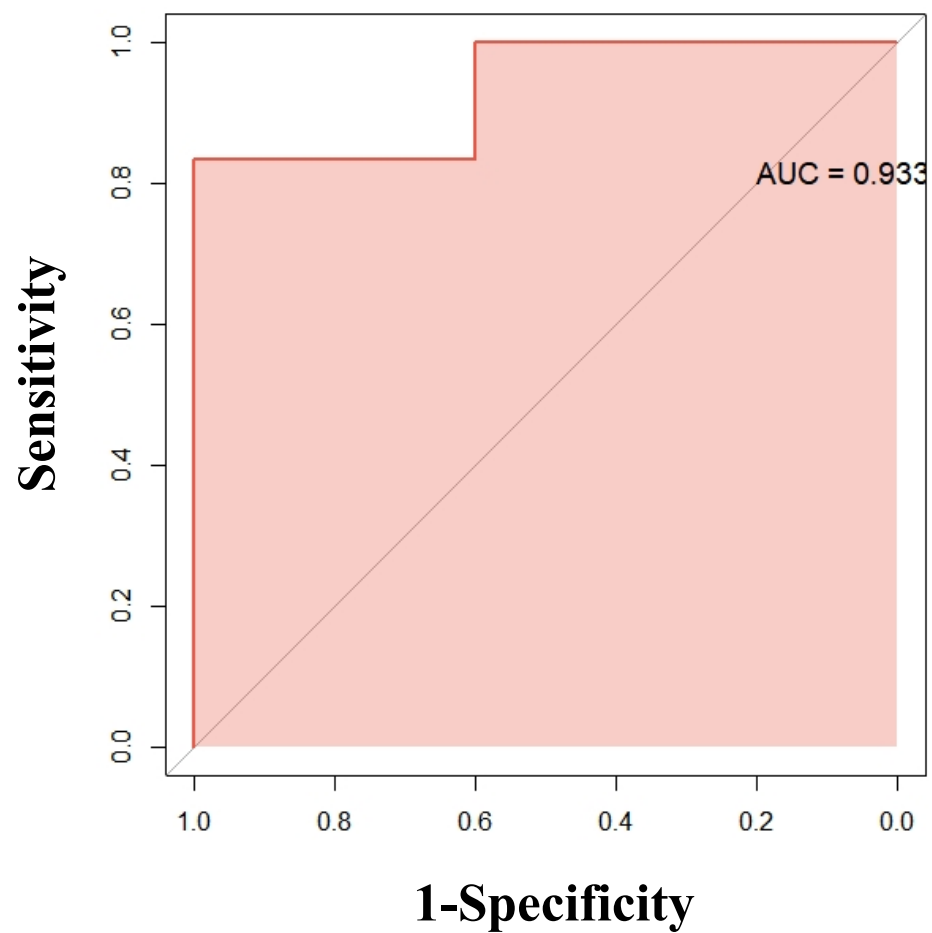

**ROC Curve for MMP9**

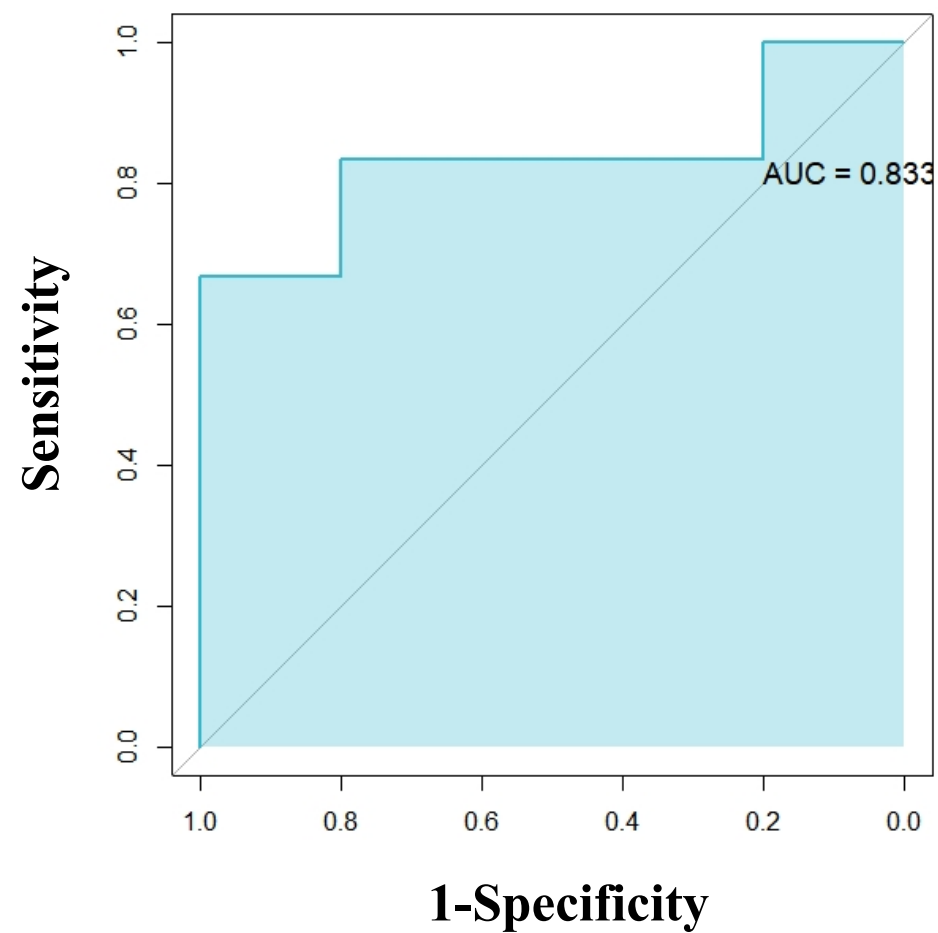

Supplement: Multimedia component 4 [file mmc4.pdf]
